# Supplementary material for: Growth, proportion, and distribution pattern of longleaf pine across southeastern forests and disturbance types: A change assessment for the period 1997-2018
Source: PLoS One. 2021 Jan 19;16(1):e0245218. doi: 10.1371/journal.pone.0245218 (PMC7815114; doi:10.1371/journal.pone.0245218)
Supplement: S1 Table — The highest-ranking values are in bold. All 1,432 plots were included in this analysis, including 36 plots that had no trees (dbh≥ 12.7 cm) either in 2004 or 2011. Where 2004 and 2011 are the centroid-year of first (1997–2010) and last (2003–2018) inventory intervals, respectively. (DOCX) [file pone.0245218.s001.docx]

**S1 Table. Stem density, basal area, and importance value percent sorted by species in 2004 and 2011.** The highest-ranking values are in bold. All 1,432 plots were included in this analysis, including 36 plots that had no trees (dbh≥ 12.7 cm) either in 2004 or 2011. Where 2004 and 2011 are the centroid-year of first (1997-2010) and second (2003-2018) inventory intervals, respectively.

| **Species** | **Density (trees ha^-1^)** | | |  | **Basal area (cm^2^ ha^-1^)** | | |  | **Importance value (%)** | | |
| --- | --- | --- | --- | --- | --- | --- | --- | --- | --- | --- | --- |
|  | **2004** | **2011** | **Change** |  | **2004** | **2011** | **Change** |  | **2004** | **2011** | **Change** |
| ***Acer barbatum*** | 0.05 | 0.05 | 0 |  | 22 | 13 | -9 |  | 0.03 | 0.03 | 0 |
| ***Acer rubrum*** | 5.39 | 5.34 | -0.05 |  | 1,544 | 1,586 | 42 |  | **2.11** | **2.17** | 0.06 |
| ***Albizia julibrissin*** |  | 0.03 | 0.03 |  |  | 6 | 6 |  |  | 0.02 | 0.02 |
| ***Carpinus caroliniana*** | 0.02 | 0.07 | 0.05 |  | 5 | 16 | 11 |  | 0.01 | 0.02 | 0.01 |
| ***Carya alba*** | 1.78 | 2.14 | 0.36 |  | 808 | 934 | 126 |  | 0.84 | 0.96 | 0.12 |
| ***Carya cordiformis*** | 0.04 |  | -0.04 |  | 9 |  | -9 |  | 0.02 |  | -0.02 |
| ***Carya glabra*** | 1.35 | 1.58 | 0.23 |  | 527 | 624 | 97 |  | 0.65 | 0.69 | 0.04 |
| ***Carya illinoinensis*** | 0.01 |  | -0.01 |  | 3 |  | -3 |  | 0.01 |  | -0.01 |
| ***Carya myristiciformis*** |  | 0.01 | 0.01 |  |  | 2 | 2 |  |  | 0.01 | 0.01 |
| ***Carya ovalis*** |  | 0.01 | 0.01 |  |  | 10 | 10 |  |  | 0.01 | 0.01 |
| ***Carya ovata*** | 0.02 |  | -0.02 |  | 9 |  | -9 |  | 0.02 |  | -0.02 |
| ***Carya pallida*** | 0.05 | 0.3 | 0.25 |  | 21 | 158 | 137 |  | 0.03 | 0.14 | 0.11 |
| ***Carya spp.*** | 0.78 |  | -0.78 |  | 237 |  | -237 |  | 0.26 |  | -0.26 |
| ***Carya texana*** | 0.12 | 0.08 | -0.04 |  | 48 | 31 | -17 |  | 0.04 | 0.02 | -0.02 |
| ***Celtis laevigata*** | 0.02 | 0.02 | 0 |  | 5 | 4 | -1 |  | 0.01 | 0.01 | 0 |
| ***Celtis occidentalis*** | 0.01 | 0.01 | 0 |  | 4 | 4 | 0 |  | 0.01 | 0.01 | 0 |
| ***Chamaecyparis thyoides*** | 0.57 | 0.42 | -0.15 |  | 382 | 272 | -110 |  | 0.21 | 0.15 | -0.06 |
| ***Cinnamomum camphora*** | 0.03 | 0.03 | 0 |  | 6 | 12 | 6 |  | 0.01 | 0.01 | 0 |
| ***Cornus florida*** | 2.86 | 1.17 | -1.69 |  | 605 | 236 | -369 |  | 1.24 | 0.62 | -0.62 |
| ***Crataegus crus-galli*** | 0.01 | 0.01 | 0 |  | 2 | 2 | 0 |  | 0.01 | 0.01 | 0 |
| ***Crataegus spp.*** |  | 0.02 | 0.02 |  |  | 6 | 6 |  |  | 0.01 | 0.01 |
| ***Diospyros virginiana*** | 0.36 | 0.25 | -0.11 |  | 82 | 52 | -30 |  | 0.2 | 0.14 | -0.06 |
| ***Fagus grandifolia*** | 0.09 | 0.12 | 0.03 |  | 36 | 55 | 19 |  | 0.06 | 0.08 | 0.02 |
| ***Family Arecaceae*** | 0.01 |  | -0.01 |  | 8 |  | -8 |  | 0.01 |  | -0.01 |
| ***Fraxinus americana*** |  | 0.01 | 0.01 |  |  | 1 | 1 |  |  | 0.01 | 0.01 |
| ***Fraxinus caroliniana*** | 0.01 | 0.01 | 0 |  | 1 | 2 | 1 |  | 0.01 | 0.01 | 0 |
| ***Fraxinus pennsylvanica*** | 0.05 | 0.05 | 0 |  | 17 | 17 | 0 |  | 0.02 | 0.02 | 0 |
| ***Gordonia lasianthus*** | 0.69 | 0.8 | 0.11 |  | 193 | 216 | 23 |  | 0.23 | 0.25 | 0.02 |
| ***Ilex opaca*** | 0.96 | 1.15 | 0.19 |  | 201 | 283 | 82 |  | 0.42 | 0.5 | 0.08 |
| ***Juglans nigra*** | 0.01 | 0.01 | 0 |  | 13 | 14 | 1 |  | 0.01 | 0.01 | 0 |
| ***Juniperus virginiana*** | 0.21 | 0.29 | 0.08 |  | 90 | 122 | 32 |  | 0.13 | 0.16 | 0.03 |
| ***Liquidambar styraciflua*** | **7.27** | **6.74** | -0.53 |  | **2,571** | **2,428** | -143 |  | **2.88** | **2.74** | -0.14 |
| ***Liriodendron tulipifera*** | 2.62 | 2.94 | 0.32 |  | 1,459 | 1,834 | 375 |  | 1.23 | 1.43 | 0.2 |
| ***Magnolia acuminata*** |  | 0.01 | 0.01 |  |  | 5 | 5 |  |  | 0.01 | 0.01 |
| ***Magnolia grandiflora*** | 0.35 | 0.62 | 0.27 |  | 127 | 225 | 98 |  | 0.18 | 0.33 | 0.15 |
| ***Magnolia macrophylla*** | 0.07 | 0.06 | -0.01 |  | 14 | 15 | 1 |  | 0.04 | 0.03 | -0.01 |
| ***Magnolia virginiana*** | **5.68** | **6.07** | 0.39 |  | 2,026 | 2,185 | 159 |  | 1.95 | **2.02** | 0.07 |
| ***Melia azedarach*** | 0.05 | 0.05 | 0 |  | 8 | 14 | 6 |  | 0.03 | 0.03 | 0 |
| ***Morus rubra*** | 0.01 | 0.01 | 0 |  | 3 | 3 | 0 |  | 0.01 | 0.01 | 0 |
| ***Morus spp.*** | 0.01 |  | -0.01 |  | 2 |  | -2 |  | 0.01 |  | -0.01 |
| ***Nyssa aquatica*** | 0.21 | 0.04 | -0.17 |  | 69 | 7 | -62 |  | 0.06 | 0.01 | -0.05 |
| ***Nyssa biflora*** | **6.15** | **6.07** | -0.08 |  | **2,281** | **2,394** | 113 |  | 1.99 | 1.97 | -0.02 |
| ***Nyssa ogeche*** |  | 0.2 | 0.2 |  |  | 76 | 76 |  |  | 0.05 | 0.05 |
| ***Nyssa sylvatica*** | 4.8 | **5.79** | 0.99 |  | 1,610 | 2,007 | 397 |  | **2.11** | **2.42** | 0.31 |
| ***Ostrya virginiana*** | 0.04 | 0.03 | -0.01 |  | 13 | 6 | -7 |  | 0.02 | 0.02 | 0 |
| ***Oxydendrum arboreum*** | 1.77 | 1.71 | -0.06 |  | 441 | 475 | 34 |  | 0.71 | 0.65 | -0.06 |
| ***Persea borbonia*** | 0.58 | 0.58 | 0 |  | 140 | 178 | 38 |  | 0.31 | 0.29 | -0.02 |
| ***Pinus clausa*** | 4.54 | 3.39 | -1.15 |  | 1,691 | 1,428 | -263 |  | 1.21 | 0.95 | -0.26 |
| ***Pinus echinata*** | 5.38 | 3.42 | -1.96 |  | **2,487** | 1,702 | -785 |  | 2.1 | 1.51 | -0.59 |
| ***Pinus elliottii*** | **35.3** | **29.89** | -5.38 |  | **16,043** | **15,724** | -319 |  | **10.7** | **9.43** | -1.27 |
| ***Pinus glabra*** | 0.12 | 0.08 | -0.04 |  | 58 | 53 | -5 |  | 0.07 | 0.06 | -0.01 |
| ***Pinus palustris*** | **70.7** | **82.19** | 11.52 |  | **39,231** | **43,692** | 4461 |  | **25.82** | **26.6** | 0.77 |
| ***Pinus serotina*** | 3.98 | 4.45 | 0.47 |  | 1,719 | **2,227** | 508 |  | 1.34 | 1.49 | 0.15 |
| ***Pinus strobus*** | 0.01 |  | -0.01 |  | 4 |  | -4 |  | 0.01 |  | -0.01 |
| ***Pinus taeda*** | **56.9** | **62.94** | 6.09 |  | **24,818** | **29,491** | 4673 |  | **16.66** | **18** | 1.29 |
| ***Pinus virginiana*** | 0.52 | 0.83 | 0.31 |  | 231 | 317 | 86 |  | 0.26 | 0.28 | 0.02 |
| ***Platanus occidentalis*** | 0.03 | 0.04 | 0.01 |  | 23 | 27 | 4 |  | 0.03 | 0.03 | 0 |
| ***Prunus serotina*** | 1.79 | 2.1 | 0.31 |  | 465 | 506 | 41 |  | 0.99 | 1.06 | 0.07 |
| ***Prunus spp.*** | 0.01 |  | -0.01 |  | 3 |  | -3 |  | 0.01 |  | -0.01 |
| ***Quercus alba*** | 1.68 | 1.37 | -0.31 |  | 865 | 848 | -17 |  | 0.88 | 0.74 | -0.14 |
| ***Quercus coccinea*** | 0.54 | 0.48 | -0.06 |  | 303 | 360 | 57 |  | 0.28 | 0.28 | 0 |
| ***Quercus falcata*** | **6.86** | 5.61 | -1.25 |  | **3,590** | **3,242** | -348 |  | **3.25** | **2.7** | -0.55 |
| ***Quercus incana*** | 0.72 | 0.53 | -0.19 |  | 142 | 136 | -6 |  | 0.38 | 0.29 | -0.09 |
| ***Quercus laevis*** | **7.73** | **5.68** | -2.05 |  | 2,111 | 1,531 | -580 |  | **2.61** | 1.97 | -0.64 |
| ***Quercus laurifolia*** | **7.5** | **9.78** | 2.28 |  | **2,768** | **4,007** | 1239 |  | **2.78** | **3.43** | 0.65 |
| ***Quercus margarettiae*** | 1.99 | 1.95 | -0.04 |  | 648 | 473 | -175 |  | 0.69 | 0.65 | -0.04 |
| ***Quercus marilandica*** | 3.5 | 2.19 | -1.31 |  | 1,150 | 741 | -409 |  | 1.37 | 0.89 | -0.48 |
| ***Quercus michauxii*** | 0.04 | 0.03 | -0.01 |  | 14 | 26 | 12 |  | 0.01 | 0.02 | 0.01 |
| ***Quercus minima*** | 1.49 | 2.19 | 0.7 |  | 399 | 614 | 215 |  | 0.46 | 0.63 | 0.17 |
| ***Quercus muehlenbergii*** | 0.02 |  | -0.02 |  | 3 |  | -3 |  | 0.01 |  | -0.01 |
| ***Quercus nigra*** | **10.1** | **12.76** | 2.62 |  | **3,858** | **4,720** | 862 |  | **4.11** | **4.86** | 0.75 |
| ***Quercus pagoda*** | 0.11 | 0.1 | -0.01 |  | 86 | 74 | -12 |  | 0.08 | 0.07 | -0.01 |
| ***Quercus phellos*** | 0.55 | 0.33 | -0.22 |  | 201 | 163 | -38 |  | 0.2 | 0.16 | -0.04 |
| ***Quercus prinus*** | 0.91 | 1.38 | 0.47 |  | 523 | 884 | 361 |  | 0.37 | 0.52 | 0.15 |
| ***Quercus rubra*** | 0.19 | 0.21 | 0.02 |  | 107 | 109 | 2 |  | 0.13 | 0.12 | -0.01 |
| ***Quercus shumardii*** | 0.02 | 0.01 | -0.01 |  | 4 | 2 | -2 |  | 0.01 | 0.01 | 0 |
| ***Quercus sinuata*** | 0.01 |  | -0.01 |  | 2 |  | -2 |  | 0.01 |  | -0.01 |
| ***Quercus stellata*** | 4.26 | 4.04 | -0.22 |  | 1,602 | 1,750 | 148 |  | 1.91 | 1.76 | -0.15 |
| ***Quercus velutina*** | 1.04 | 0.81 | -0.23 |  | 400 | 451 | 51 |  | 0.46 | 0.45 | -0.01 |
| ***Quercus virginiana*** | 3.51 | 4.51 | 1 |  | **2,154** | **2,603** | 449 |  | 1.54 | 1.81 | 0.27 |
| ***Sabal palmetto*** | 0.17 | 0.31 | 0.14 |  | 141 | 223 | 82 |  | 0.08 | 0.13 | 0.05 |
| ***Salix spp.*** | 0.02 |  | -0.02 |  | 5 |  | -5 |  | 0.01 |  | -0.01 |
| ***Sassafras albidum*** | 0.16 | 0.16 | 0 |  | 37 | 32 | -5 |  | 0.09 | 0.1 | 0.01 |
| ***Taxodium ascendens*** | 1.88 | 2.11 | 0.23 |  | 883 | 1,053 | 170 |  | 0.6 | 0.67 | 0.07 |
| ***Taxodium distichum*** | 0.43 | 0.17 | -0.26 |  | 141 | 92 | -49 |  | 0.14 | 0.06 | -0.08 |
| ***Tree unknown*** | 0.07 |  | -0.07 |  | 14 |  | -14 |  | 0.02 |  | -0.02 |
| ***Triadica sebifera*** | 0.23 | 0.18 | -0.05 |  | 71 | 55 | -16 |  | 0.07 | 0.08 | 0.01 |
| ***Ulmus alata*** | 0.06 | 0.18 | 0.12 |  | 14 | 42 | 28 |  | 0.04 | 0.1 | 0.06 |
| ***Ulmus americana*** | 0.12 | 0.06 | -0.06 |  | 42 | 29 | -13 |  | 0.05 | 0.03 | -0.02 |
| ***Ulmus rubra*** |  | 0.01 | 0.01 |  |  | 12 | 12 |  |  | 0.01 | 0.01 |
| ***Ulmus spp.*** | 0.03 |  | -0.03 |  | 6 |  | -6 |  | 0.02 |  | -0.02 |
