# Supplementary material for: Growth, proportion, and distribution pattern of longleaf pine across southeastern forests and disturbance types: A change assessment for the period 1997-2018
Source: PLoS One. 2021 Jan 19;16(1):e0245218. doi: 10.1371/journal.pone.0245218 (PMC7815114; doi:10.1371/journal.pone.0245218)
Supplement: S2 Table — (DOCX) [file pone.0245218.s002.docx]

**S2 Table. Estimates from linear mixed models examining longleaf pine basal area ratio (N =1,432).**

|  | **Estimates** | | | |
| --- | --- | --- | --- | --- |
|  | **model 1** | **model 2** | **model 3** | **model 4** |
| **Fixed-effects** |  |  |  |  |
| **Intercept** | 0.36^*^ (0.01) | 0.36^*^ (0.009) | 0.36^*^ (0.0086) | 0.35^*^ (0.01) |
| **Time (year)** |  | -0.001^δ^ (0.0004) | -0.001^β^ (0.0004) | -0.001^γ^ (0.0004) |
| **Plot condition** |  |  |  |  |
| **C** |  |  |  | -0.02 (0.02) |
| **F** |  |  |  | 0.16^*^ (0.03) |
| **FC** |  |  |  | 0.04 (0.04) |
| **OTH** |  |  |  | -0.06 (0.04) |
| **W** |  |  |  | -0.09^§^ (0.05) |
| **WC** |  |  |  | -0.07 (0.06) |
| **ND** |  |  |  | 0 |
| **Covariance parameter (error variance)** |  |  |  |  |
| **Residual** | 0.02^*^ (0.001) | 0.02^*^ (0.0009) | 0.01^*^ (0.001) | 0.01^*^ (0.001) |
| **Intercept** | 0.09^*^ (0.004) | 0.09^*^ (0.004) | 0.09^*^ (0.004) | 0.09^*^ (0.004) |
| **Time (year)** |  |  | 0.0001^*^ (0.00001) | 0.0001^*^ (0.00002) |
| **Model fit** |  |  |  |  |
| **AIC** | 521.7 | 517.8 | 457.6 | 421.1 |
| **BIC** | 537.5 | 538.9 | 484.0 | 479.3 |
| Note: Statistically significant, ^*^*p* <0.0001, ^δ^*p*=0.01, ^β^*p*=0.007, ^γ^*p*=0.008, ^§^*p*=0.04; Intraclass Correlation Coefficient (ICC) = 0.79 | | | | |
| Values are based on SAS PROC Mixed. Parameter estimates of variables are presented with standard errors in parentheses. Estimation Method = Maximum likelihood (ML); Satterthwaite degrees of freedom. Time is the plot remeasurement period in year. | | | | |
| Plot condition codes are described in Table 1 | | | | |
