# Supplementary material for: Growth, proportion, and distribution pattern of longleaf pine across southeastern forests and disturbance types: A change assessment for the period 1997-2018
Source: PLoS One. 2021 Jan 19;16(1):e0245218. doi: 10.1371/journal.pone.0245218 (PMC7815114; doi:10.1371/journal.pone.0245218)
Supplement: S3 Table — (DOCX) [file pone.0245218.s003.docx]

**S3 Table. Estimates from linear mixed models examining longleaf pine basal area (m^2^ ha^-1^) (N =1,432).**

|  | **Estimates** | | | | | | |
| --- | --- | --- | --- | --- | --- | --- | --- |
|  | **model 1** | **model 2** | | **model 3** | | **model 4** | |
| **Fixed-effects** |  | |  | |  | |  |
| **Intercept** | 4.0^*^ (0.13) | | 3.9^*^ (0.13) | | 3.9^*^ (0.13) | | 4.0^*^ (0.17) |
| **Time (year)** |  | | 0.03^*^ (0.007) | | 0.03^*^ (0.008) | | 0.03^*^ (0.007) |
| **Plot condition** |  | |  | |  | |  |
| **C** |  | |  | |  | | -1.1^δ^ (0.31) |
| **F** |  | |  | |  | | 2.2^*^ (0.40) |
| **FC** |  | |  | |  | | 0.2 (0.67) |
| **OTH** |  | |  | |  | | -1.0 (0.66) |
| **W** |  | |  | |  | | -0.6 (0.67) |
| **WC** |  | |  | |  | | -1.5 (0.85) |
| **ND** |  | |  | |  | | 0 |
| **Covariance parameter (error variance)** |  | |  | |  | |  |
| **Residual** | 5.6^*^ (0.21) | | 5.6^*^ (0.21) | | 2.09^*^ (0.21) | | 2.10^*^ (0.22) |
| **Intercept** | 20.5^*^ (0.87) | | 20.4^*^ (0.87) | | 21.01^*^ (0.86) | | 20.10^*^ (0.82) |
| **Time (year)** |  | |  | | 0.037^*^ (0.003) | | 0.037^*^ (0.003) |
| **Model fit** |  | |  | |  | |  |
| **AIC** | 16,118.0 | | 16,094.9 | | 15,916.7 | | 15,871.9 |
| **BIC** | 16,133.8 | | 16,116.1 | | 15,943.1 | | 15,930.1 |
| Note: Statistically significant, ^*^*p*<0.0001, ^δ^*p*=0.0004; Intraclass Correlation Coefficient (ICC) = 0.78 | | | | | | | |
| Values are based on SAS PROC Mixed. Parameter estimates of variables are presented with standard errors in parentheses. Estimation Method = Maximum likelihood (ML); Satterthwaite degrees of freedom. Time is the plot remeasurement period in year. | | | | | | | |

Plot condition codes are described in Table 1
