# Supplementary material for: Growth, proportion, and distribution pattern of longleaf pine across southeastern forests and disturbance types: A change assessment for the period 1997-2018
Source: PLoS One. 2021 Jan 19;16(1):e0245218. doi: 10.1371/journal.pone.0245218 (PMC7815114; doi:10.1371/journal.pone.0245218)
Supplement: Attachment — Submitted filename: Response to Reviewers.docx [file pone.0245218.s004.docx]

**Responses are in bold**

Additional Editor Comments:

This is a descriptive study of changes taking place across longleaf pine ecosystems in SE USA. This information could be relevant for conservation of these systems and as guidance for future research projects aimed at identifying and quantifying agents of change. The study is sound and well written but needs a few clarifications.

Years used in the analysis should be specified clearly, title mentions 1997-2018, while analysis refers to 2004 and 2011 centroids of 1997-2010 and 2003-2018 intervals, make clear what it was used/done specially because the intervals overlap and it is not clear if plots were counted twice.

**We clarified this point in the Methods section, including with this statement: “The overlap in the two periods, 1997-2010 and 2003-2018, means that some of the plots were getting measured a second time, while others have not yet been measured for the first time.” So every plot was counted only once for each interval.**

My suggestion would be to use the time intervals, e.g., 1-22 years, rather than actual years.

**We felt important to make it clear when the first and last measurement were carried out between the years specified, hence our preference to list the range of years for the first and range of years for the last interval.**

Also, the intervals between censuses was quite different 1 to 22 years, changes should be standardized per year basis (something done in the analysis but rate of change is likely not linear, e.g., much higher after disturbance).

**The growth (AGB) was indeed standardized per year. And while trees certainly do not have linear growth over their lifetime, though for shorter periods of time, such as ours, this is not as much of an issue.**

Methods, L230 and beyond. It is not clear if the analysis is based on FAI data solely and the IDW was only for the purpose of visualizing results at sites not included in the analysis; or if there was a second analysis of remote sensing data. Please, clarify. If the latest, I can see interpolating biomass, but I’m not sure that would work for diversity, please, explain what it was done and its feasibility.

**It is the former: we used the FIA data and the IDW to estimate values at other locations. We now added this clarification in the text.**

L420-422 what does actually mean?

**We now clarified that the 0.79 value of Intraclass Correlation Coefficient (ICC) for the response variable longleaf pine basal area ratio indicated that there was 79% variability in the longleaf pine basal area ratio among the plots. ICC is defined in the Methods section. This high variability among the plots meant that the basal area ratios were more dissimilar and extreme values were likely present across the plots.**

Results: it is not clear what is the value of showing, and commenting on, all models. My suggestion is to only mention, and comment on, model with best fit (model 4), and have those tables moved to the supplement.

**Changed as per suggestion. Tables moved to supplement.**

For all the variables and ratios measured indicated what changes on its values would indicate about those sites, e.g., natural successional dynamics, degradation, changes in community composition, …**We addressed this in Discussion section and improved the manuscript.**

Journal Requirements:

When submitting your revision, we need you to address these additional requirements.

 1. Please ensure that your manuscript meets PLOS ONE's style requirements, including those for file naming. The PLOS ONE style templates can be found at

<https://journals.plos.org/plosone/s/file?id=wjVg/PLOSOne_formatting_sample_main_body.pdf> and

<https://journals.plos.org/plosone/s/file?id=ba62/PLOSOne_formatting_sample_title_authors_affiliations.pdf>

2. We note that Figures 1, 2, 6, 7 in your submission contain map images which may be copyrighted. All PLOS content is published under the Creative Commons Attribution License (CC BY 4.0), which means that the manuscript, images, and Supporting Information files will be freely available online, and any third party is permitted to access, download, copy, distribute, and use these materials in any way, even commercially, with proper attribution. For these reasons, we cannot publish previously copyrighted maps or satellite images created using proprietary data, such as Google software (Google Maps, Street View, and Earth). For more information, see our copyright guidelines: <http://journals.plos.org/plosone/s/licenses-and-copyright>.

**We used US state boundary shapefiles in Figures 1, 2, 6, 7 from the website of the US Census Bureau, which is freely available to download and public use. We cited this source** (**US Census Bureau, 2020).**

**The Longleaf pine range boundary shapefile was downloaded from the website of the USDA Natural Resources Conservation Service (NRCS)** **and used** **in Figures 1, 2, 6, 7, which is also publicly available to use. We also cited this source (NRCS, 2020).**

**The shapefile of the physiographic divisions of the conterminous U. S. was downloaded from the website of the US Geological Survey (**[**https://water.usgs.gov**](https://water.usgs.gov)**) and used in Figures 1, 2, 6, 7, which is also freely available to use with a citation. We also cited this source (Fenneman, N.M., Johnson, D.W., 1946).**

We require you to either (1) present written permission from the copyright holder to publish these figures specifically under the CC BY 4.0 license, or (2) remove the figures from your submission:

2.1.    You may seek permission from the original copyright holder of Figures 1, 2, 6, 7 to publish the content specifically under the CC BY 4.0 license.

**We used only freely and publicly available shapefile images from government agencies. We cited all of them.**

We recommend that you contact the original copyright holder with the Content Permission Form (<http://journals.plos.org/plosone/s/file?id=7c09/content-permission-form.pdf>) and the following text:

“I request permission for the open-access journal PLOS ONE to publish XXX under the Creative Commons Attribution License (CCAL) CC BY 4.0 (<http://creativecommons.org/licenses/by/4.0/>). Please be aware that this license allows unrestricted use and distribution, even commercially, by third parties. Please reply and provide explicit written permission to publish XXX under a CC BY license and complete the attached form.”

Please upload the completed Content Permission Form or other proof of granted permissions as an "Other" file with your submission.

In the figure caption of the copyrighted figure, please include the following text: “Reprinted from [ref] under a CC BY license, with permission from [name of publisher], original copyright [original copyright year].”

2.2.    If you are unable to obtain permission from the original copyright holder to publish these figures under the CC BY 4.0 license or if the copyright holder’s requirements are incompatible with the CC BY 4.0 license, please either i) remove the figure or ii) supply a replacement figure that complies with the CC BY 4.0 license. Please check copyright information on all replacement figures and update the figure caption with source information. If applicable, please specify in the figure caption text when a figure is similar but not identical to the original image and is therefore for illustrative purposes only.

**Answered above.**

The following resources for replacing copyrighted map figures may be helpful:

USGS National Map Viewer (public domain): <http://viewer.nationalmap.gov/viewer/>

The Gateway to Astronaut Photography of Earth (public domain): <http://eol.jsc.nasa.gov/sseop/clickmap/>

Maps at the CIA (public domain): <https://www.cia.gov/library/publications/the-world-factbook/index.html> and <https://www.cia.gov/library/publications/cia-maps-publications/index.html>

NASA Earth Observatory (public domain): <http://earthobservatory.nasa.gov/>

Landsat: <http://landsat.visibleearth.nasa.gov/>

USGS EROS (Earth Resources Observatory and Science (EROS) Center) (public domain): [http://eros.usgs.gov/#](http://eros.usgs.gov/)

Natural Earth (public domain): <http://www.naturalearthdata.com/>
